# Identification of EvenBluerMoon: a bacteriophage of *Arthrobacter globiformis* from Lubbock, Texas, topsoil

**DOI:** 10.1128/mra.00638-25

**Published:** 2025-09-11

**Authors:** Laurissa N. Miller, Natalie Block, Whitney Dickens, Carson Bellew, Christian Deluna, Francesca Makilan, Malli Bhakta, Ashleigh Crawford, Trinity Criner, Chase Drucker, Aqsa Fayyaz, Jasmine Goh, Caitlyn Guetersloh, Claire Jansen, Dana Pham, Andrea Resendez, Austen Rowell, Fahareen B. Mosharraf, Allie C. Smith, Lisa M. Bono

**Affiliations:** 1 Department of Biological Sciences, Texas Tech University 6177, Lubbock, Texas, USA; 2 Teaching, Learning, and Professional Development Center, University Library, Texas Tech University 6177, Lubbock, Texas, USA; 3 TrUE Scholars Program, Center for Transformative Undergraduate Experiences, Texas Tech University 6177, Lubbock, Texas, USA; 4 Honors College, Texas Tech University 6177, Lubbock, Texas, USA; Portland State University, Portland, Oregon, USA

**Keywords:** bacteriophage, EvenBluerMoon, *Arthrobacter globiformis*, *Arthrobacter* spp., SEA-PHAGES, genome sequencing, environmental phage, soil collection

## Abstract

We report the genome sequence analysis of an environmental bacteriophage discovered from topsoil in Lubbock, Texas, USA, as part of the SEA-PHAGES program. The bacteriophage, EvenBluerMoon, infects *Arthrobacter globiformis* and is a member of the FO cluster. EvenBluerMoon has an icosahedral head with a long non-contractile tail.

## ANNOUNCEMENT

*Arthrobacter* spp. are rod-shaped, gram-positive bacteria that can be commonly found in soil (1). Through the Science Education Alliance-Phage Hunters Advancing Genomics and Evolutionary Sciences (SEA-PHAGES) program, we used *Arthrobacter globiformis* B-2979 (accession number AF329477.3) (2) as a host to isolate, sequence, and characterize environmental bacteriophage from Lubbock, Texas, USA.

The EvenBluerMoon bacteriophage (3) was collected from a soil sample (1–5 cm in depth) on 30 August 2023 at coordinates 33.58482, –101.87825. In a 50 mL Falcon tube, soil was added to the 15 mL mark, and peptone yeast calcium (PYCa) broth was then added to the 35 mL mark. The tube was vortexed and centrifuged at 2,000 × *g* (3,500 rpm) for 10 min. Debris and cells were removed using a 0.22 µm syringe filter. The filtrate was seeded with 500 µL culture of *A. globiformis*, incubated at 30°C at 220 rpm for 3 days, and diluted and plated using an agar-overlay method. Plates were incubated at 30°C for 48 h (4). One resulting plaque after triple plaque purification was cut out and suspended in a solution of 400 µL of PYCa broth and 400 µL of 80% glycerol (4). From this, a high titer lysate (>1 × 10^9^ pfu/mL) was produced. DNA was extracted from the lysate using a Quantabio Extracta Plus DNA Kit (Cat.# 95213-050). The quantity of extracted DNA was calculated using a BioTek Take3 plate reader. An Illumina DNA Prep tagmentation kit was used to prepare the extracted DNA for whole genome sequencing on an Illumina NextSeq 2000 platform using a 300-cycle flow cell kit, generating 2 × 150 bp paired reads.

Raw Illumina reads were trimmed, and the quality was assessed by Fastp v0.23.4 (5). Bowtie2 v2.5.3 (6) was used to remove host contamination (Table 1). The genome was assembled using SPAdes genome assembler v3.15.5 (7). SAMtools v1.6 was used to calculate sequencing depth (average number of reads per base), and BEDTools v2.31.0 was used to determine genome coverage (the proportion of the genome covered by at least one read) (8, 9). The assembled genome was annotated using DNAMaster v5.23.3 and further confirmed by Phage Evidence Collection And Annotation Network (10, 11). Prediction and manual validation of coding sequences were performed using Glimmer v3.0 and GeneMark v2.5 (12). The function of these sequences was determined by using BLAST v2.11.01 (13), HHPRED v3.0 (14) (using the PDB_mmCIF70), Pfam-v36.0 (15), NCBI Conserved Domains databases (16), and Phamerator (17) (using Actino_draft database v578). All software was used with default settings.

**TABLE 1:** Characterization of the reported bacteriophage

Parameter	Data for the isolated bacteriophage EvenBluerMoon
Genome length (bp)	36,231
Total number of reads	1,269,200
Sequencing coverage (×)	2,424
No. of predicted genes	51
No. of tRNAs	0
No. of hypothetical proteins of unknown function	29
GC content (%)	68.8%
GenBank accession no.	PV329799
SRA accession no.	SRR31925871
BioProject accession no.	PRJNA488469

**Fig 1:**
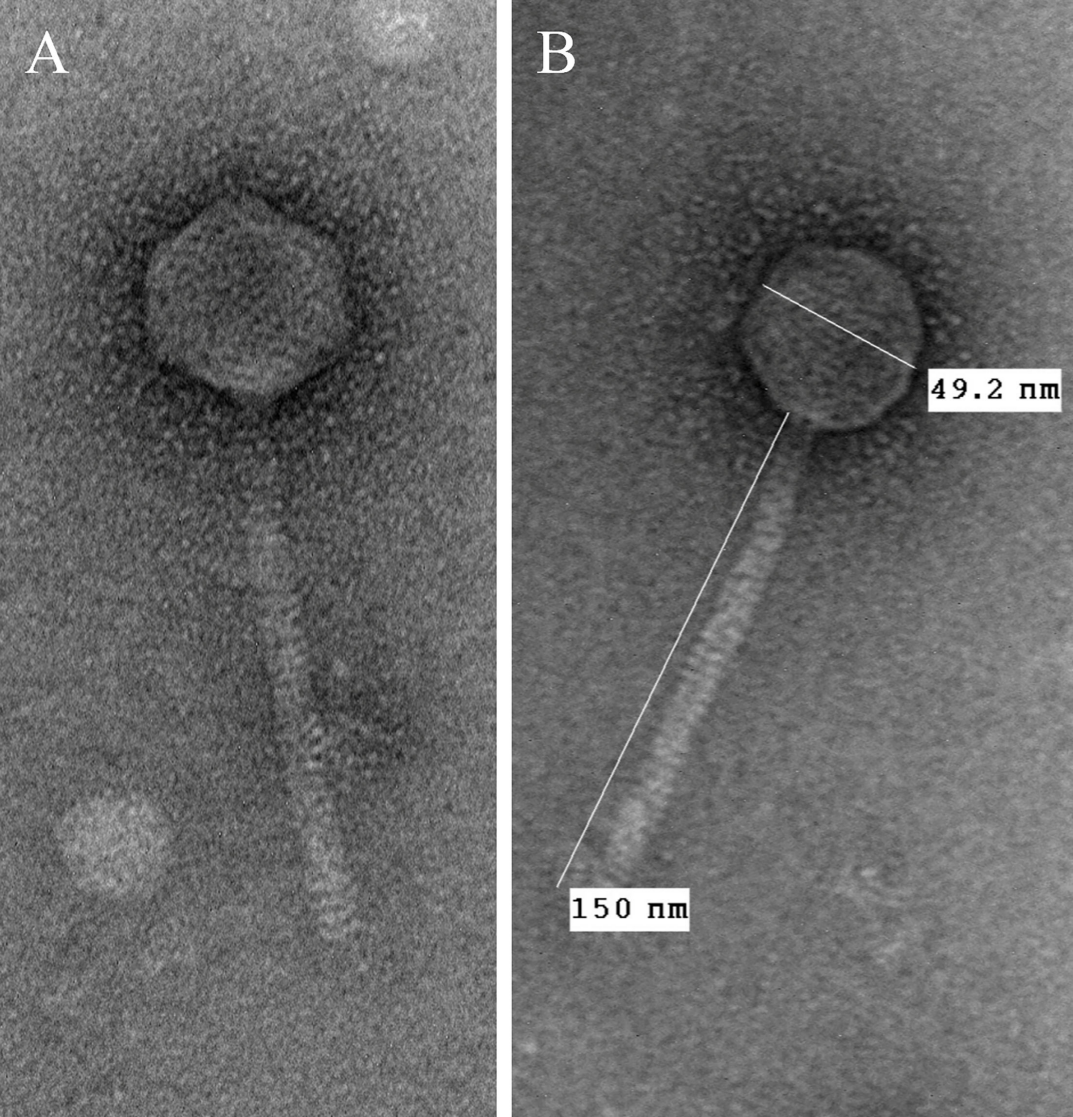
Electron micrographs of the tailed bacteriophage EvenBluerMoon (**A**) with measurements (**B**). Measurements were quantified with ImageJ.

Lysates were stained with 1% uranyl acetate and viewed using a transmission electron microscope at 100 kV (Hitachi H-7650; Hitachi, Tokyo, Japan) (Fig. 1).

EvenBluerMoon was manually classified as having 3′ sticky overhang genome termini. It is a member of the FO cluster of the Actinobacteriophage Database (PhagesDB.org). The genome includes common structural and replication-associated genes typical of lytic phages, with several genes of unknown function. No tRNAs were identified using tRNAscan-SE v2.0 and ARAGORN v1.2.38 (18, 19).

## Data Availability

This project has been deposited in GenBank under accession number PV329799 as well as the NCBI Sequence Read Archive (SRA) database under accession number SRR31925871. The BioProject accession number is PRJNA488469.
